# Milk extracellular vesicles: A burgeoning new presence in nutraceuticals and drug delivery

**DOI:** 10.1002/btm2.10756

**Published:** 2025-01-23

**Authors:** Spencer R. Marsh, Claire E. Beard, Robert G. Gourdie

**Affiliations:** ^1^ Fralin Biomedical Research Institute at VTC Virginia Tech Roanoke Virginia USA; ^2^ Center for Heart and Reparative Medicine Research Virginia Tech Roanoke Virginia USA; ^3^ The Tiny Cargo Company Roanoke Virginia USA; ^4^ Translational Biology Medicine and Health Graduate Program Virginia Tech Roanoke Virginia USA; ^5^ Department of Biomedical Engineering and Mechanics Virginia Tech Blacksburg Virginia USA; ^6^ Department of Emergency Medicine Virginia Tech Carilion School of Medicine, Virginia Tech Roanoke Virginia USA

**Keywords:** drug delivery, exosome, extracellular vesicle, infant development, milk, nutraceutical, pharmaceutical

## Abstract

Mammalian milk, a multifaceted developmental biofluid, has attracted new attention due to its diverse constituents and their implications for health and disease. Among these constituents, extracellular vesicles (EVs) have emerged as focal points of investigation. EVs, including exosomes and small EVs, have demonstrated biological activity in preclinical studies—including reports of enhancement of cognition and neural complexity, promotion of gastrointestinal development, barrier function and microbiome richness, the bolstering of immune response, and facilitation of musculoskeletal maturation in neonates. The richness of milk as a source of EVs is noteworthy, with hundreds of milliliters (at >10^12^ EVs/mL) of nanovesicles extractable from a single liter of milk (>10^14^ EVs/starting liter of milk). Techniques such as tangential flow filtration hold promise for scalable production, potentially extending to thousands of liters. Together with the scale and increasing sophistication of the dairy industry, the abundance of EVs in milk underscores their commercial potential in various nutraceutical applications. Beyond natural bioactivity, milk EVs (mEVs) present intriguing possibilities as orally deliverable, non‐immunogenic pharmaceutical carriers, with burgeoning interest in their utilization for heart disease and cancer chemotherapy and as vectors for gene‐editing modules such as CrispR. This review synthesizes current knowledge on mEV biogenesis, characterization, isolation methodologies, and cargo contents. Moreover, it delves into the therapeutic potential of mEVs, both as inherently bioactive nanovesicles and as versatile platforms for drug delivery. As efforts progress toward large‐scale implementation, rigorous attention to safe, industrial‐scale production and robust assay development will be pivotal in harnessing the translational promise of small EVs from milk.

**Translational Impact Statements**

Milk extracellular vesicles (mEVs) offer a novel, naturally occurring platform for drug delivery, capable of transporting potent therapeutics such as small molecules, peptides, and gene‐editing agents in a non‐immunogenic, orally deliverable format. Their unique properties enable them to survive gastrointestinal conditions and effectively cross biological barriers, making them ideal candidates for oral administration. Additionally, the native bioactive cargoes of mEVs present an abundant source of nutraceuticals, with potential applications ranging from gastrointestinal microbiome support to tissue regeneration. This review highlights the potential of mEVs and emphasizes the urgent need for safe commercial‐scale production of mEVs vesicles to facilitate their translation into therapeutic and nutraceutical applications.

## 1 INTRODUCTION

Extracellular vesicles (EVs) represent a ubiquitous class of nanoscale lipid‐bilayer vesicles secreted by cells that are presently under intensive study across a variety of biological contexts. These vesicles have been isolated from diverse sources, including animal and plant tissues, cell culture medium, plants, blood, and a range of other bodily fluids from urine to sweat.^1^, ^2^, ^3^, ^4^ Remarkably, EVs serve as crucial mediators of intercellular communication among both eukaryotic and prokaryotic cells, underscoring their fundamental role in biological processes.^5^ Notably, EVs are not known to be generated by viruses, many of which fall in a similar size range to EVs. The ubiquity of EVs across living systems positions them as key players in communication networks between cells of all type.

Whilst EVs are copiously present in nearly all biological fluids, as well as the media of cultured cells, milk provides one of the richest sources of these nanovesicles, with hundreds of milliliters of EVs extractable from a single liter of milk at ultradense concentration—in excess of 10^12^ mEVs/milliliter.^1^ Techniques such as tangential flow filtration (TFF) hold promise for realizing industrial‐scale production, potentially extending to thousands of liters. With the growing evidence of mEVs in brain development,^6^, ^7^, ^8^, ^9^, ^10^ gastrointestinal health,^11^, ^12^, ^13^, ^14^, ^15^, ^16^, ^17^, ^18^, ^19^, ^20^, ^21^, ^22^ immune function,^23^, ^24^, ^25^, ^26^, ^27^, ^28^ and tissue repair and regeneration,^29^, ^30^, ^31^, ^32^, ^33^ as well as the potential of these nanovesicles as non‐immunogenic vehicles for drug delivery,^34^, ^35^, ^36^, ^37^, ^38^, ^39^, ^40^, ^41^, ^42^ the abundance of mEVs in milk underscores their clinical and industrial potential.^6^, ^7^, ^8^, ^9^, ^10^, ^11^, ^12^, ^13^, ^14^, ^15^, ^16^, ^17^, ^18^, ^19^, ^20^, ^21^, ^22^, ^23^, ^24^, ^25^, ^26^, ^27^, ^28^, ^29^, ^30^, ^31^, ^32^, ^33^, ^34^, ^35^, ^36^, ^37^, ^38^, ^39^, ^40^, ^41^, ^42^, ^43^, ^44^, ^45^, ^46^, ^47^, ^48^, ^49^, ^50^, ^51^, ^52^, ^53^, ^54^, ^55^

**FIGURE 1:**
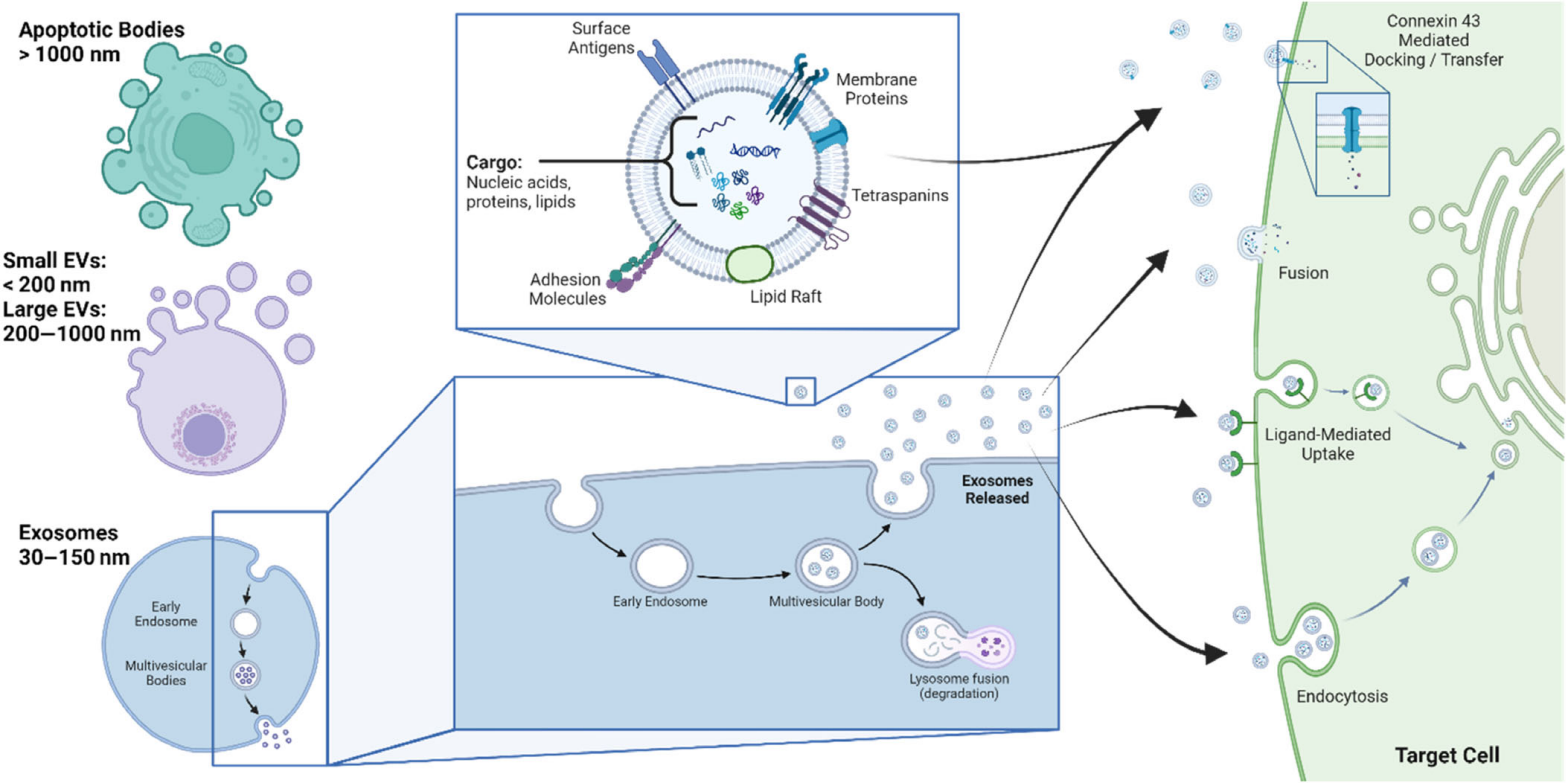
Overview of extracellular vesicle (EV) biogenesis (left), composition (center), and uptake (right). Created with Biorender.com.

In this review we survey data underlining this potential, but before doing so we will first outline some basic definitions and approaches used in the EV field. EVs encompass various membrane‐bound vesicles, including exosomes, microvesicles, and apoptotic bodies—illustrated in Figure 1.^56^ The smallest category, known as exosomes, are the most commonly associated with biological effects. These are typically classified as EVs with a diameter <150 nm, with proteomic and other standard characterizations as has been detailed by the International Society for Extracellular Vesicles.^56^ Exosomes are a sub‐class of small EVs that are formed by the Endosomal Sorting Complex Required for Transport (ESCRT) machinery, which has been reviewed extensively by others.^57^, ^58^, ^59^

The next category historically was referred to as “microvesicles,” yet this term has fallen out of broad use, in preference for designations that include “small EVs” (<200 nm) and “large EVs” (200—1000 nm).^60^, ^61^ The size range of small EVs obviously presents ambiguity with respect to exosomes; however, the biogenesis of this type of small EV is distinct, with this class of EV being formed by budding from the cell membrane rather than the ESCRT pathway.^62^ This being said, the evidence for a role of small and large EVs in intercellular communication seems to be just as profound as their exosomal counterparts.^63^ Most isolation procedures fail to differentiate exosomes of “small EVs” type, resulting in EV yields of <200 nm vesicles typically comprising those of both exosomal and “microvesicular” (or small EV) origin.^1^

The last category of EVs is presently considered to be of the least direct interest in nutraceutical and pharmaceutical development, yet arguably having the largest impact on cells within the body—apoptotic bodies.^64^ These are formed during cell death and are considered to carry “pro‐death,” or bystander‐mediated cell death signals to surrounding cells and tissues. The propensity to propagate “pro‐death” signals has not stopped researchers from investigating the possibilities of apoptotic bodies in drug delivery.^65^ While this review will not touch on apoptotic bodies past this introduction due to their apparent lack of abundance in milk, the role of this class of EV in dictating tissue and cellular responses to injury and infection should not be underestimated.^66^

Uptake of EVs occurs via a variety of mechanisms, as reviewed previously^67^ and illustrated in Figure 1; direct cell fusion is a primary mechanism, as well as various forms of endocytosis, including macropinocytosis, clathrin‐ and caveolae‐mediated endocytosis, and phagocytosis.^68^, ^69^, ^70^, ^71^, ^72^ Also noted by Soares et al.,^73^ the gap junction protein Connexin‐43 (Cx43) has been implicated in EV uptake; this raises the additional possibility of connexins and other channel proteins, which have been identified in EVs, being involved in EV uptake and transduction of EV‐cargo signals.

The isolation and purification of EVs from different sources, such as serum, culture media, or tissue can be performed in a variety of ways, from highly reproducible, yet low‐yield techniques, such as ultracentrifugation,^74^ magnetic bead recovery,^75^ immune‐affinity capture^76^ and density gradient separation,^77^ to higher‐yield methodologies, including TFF^1^ and size exclusion chromatography.^78^ Prior researchers have directly compared these methods head‐to‐head,^79^ and reviews covering the different general approaches to purifying EVs have been reported by a number of groups.^80^, ^81^

The literature reveals that mEVs can also be purified using a battery of approaches. Given the complex composition of milk, including proteins such as caseins and compounds with characteristics in common with EVs present in this biofluid, such as lipoproteins, simple approaches such as ultracentrifugation are generally less adept at purifying high yields of purified EVs.^1^ Given this, many research groups opt to use a combination of techniques that increase purification efficiency and yield, incorporating methods such as TFF and size exclusion chromatography,^1^ or ultracentrifugation coupled with density gradient separation.^82^

The challenge for the future is the translation of these lab‐based mEV isolation methods to approaches for production at industrial scale. The promise of scalable production, coupled with the natural bioactivity and drug delivery potential of mEVs, highlights their potential in both the clinic and the market. This potential is further underscored by the fact that milk is one of the most widely consumed and voluminously generated products of human agriculture. The subsequent sections of this review will delve into the characterization and biology of exosomal constituents found in milk, alongside an examination of published data regarding the inherent biological activity and pharmaceutical applications of mEVs.

## 2 CHARACTERIZATION OF MILK EXTRACELLULAR VESICLES

**FIGURE 2:**
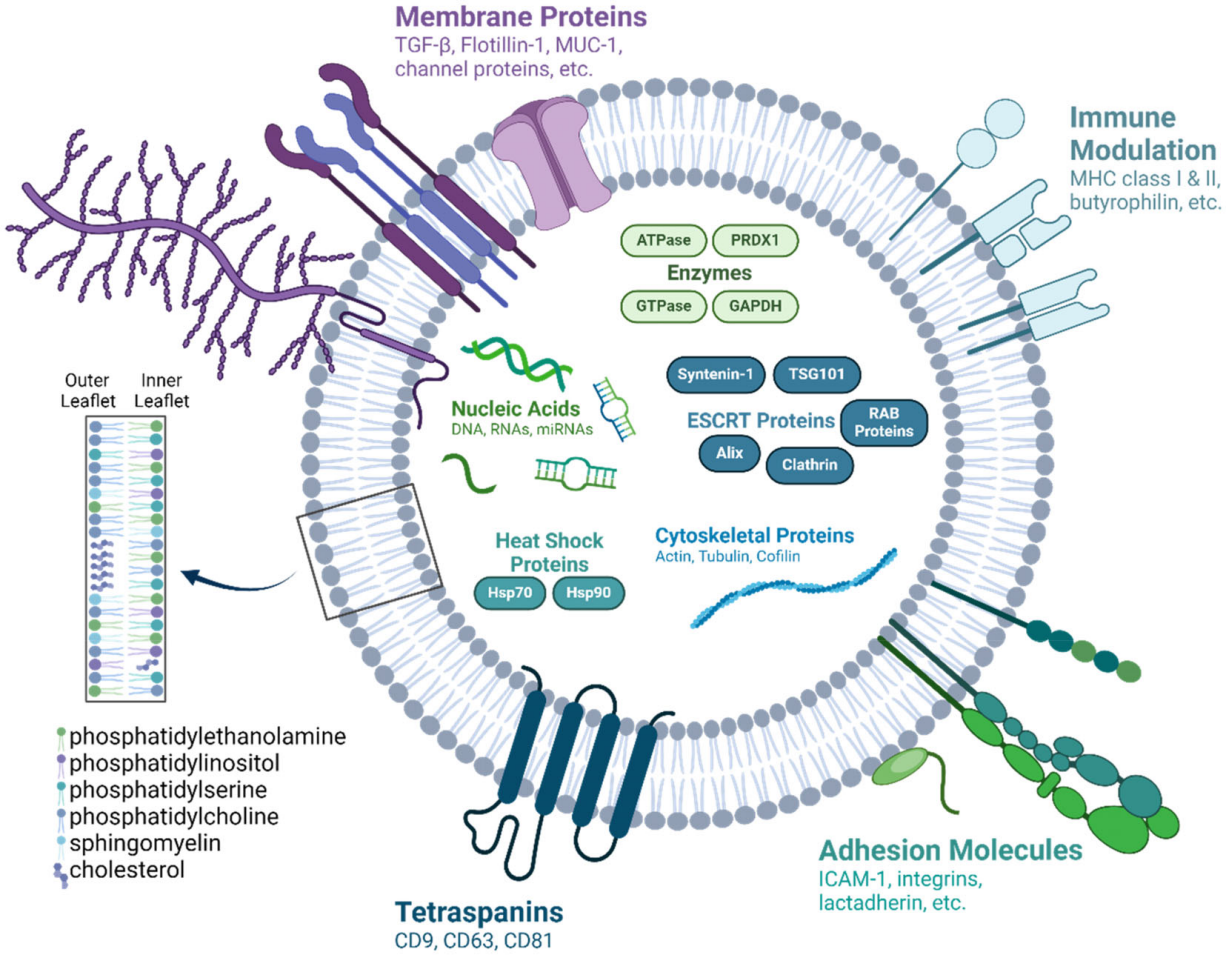
Proteomic, lipidomic, and transcriptomic composition of small milk extracellular vesicles. Created with BioRender.com.

The techniques used to characterize and classify mEVs tend to be similar to those used for small EVs from other sources. Using minimal information for study of EVs (MISEV) 2018 standards as a guide, researchers employ markers for lipids, as well as proteins—and find that the lipidomic composition of mEVs from various mammalian species tends to be very similar.^83^, ^84^ In fact, lipid compositions appear to only slightly vary between mEVs and EVs from other sources, suggesting an overall conservation of lipid composition.^85^, ^86^ Specifically, EVs are enriched in sphingolipids and glycerophospholipids, as well as phosphatidylcholine, phosphatidylserine, sphingomyelin, and cholesterol.^83^, ^84^, ^85^, ^86^ Proteins acceptable for characterization of EVs include tetraspanins (CD9, CD81, and CD63), heat shock proteins (HSP70), syntenin‐1, and tumor susceptibility gene 101 (TSG‐101).^87^, ^88^, ^89^ At least one negative marker should be used in addition to positive markers for exosomes. The most popular negative marker is Calnexin, an endoplasmic reticulum protein that is also found in apoptotic bodies, but not in exosomes or small EVs.^11^ A comprehensive review of the protein and lipid content of mEVs was published in 2023 by Buratta et al.^83^ The most common EV proteins and lipids, as well as cargoes, are summarized in Figure 2.

**FIGURE 3:**
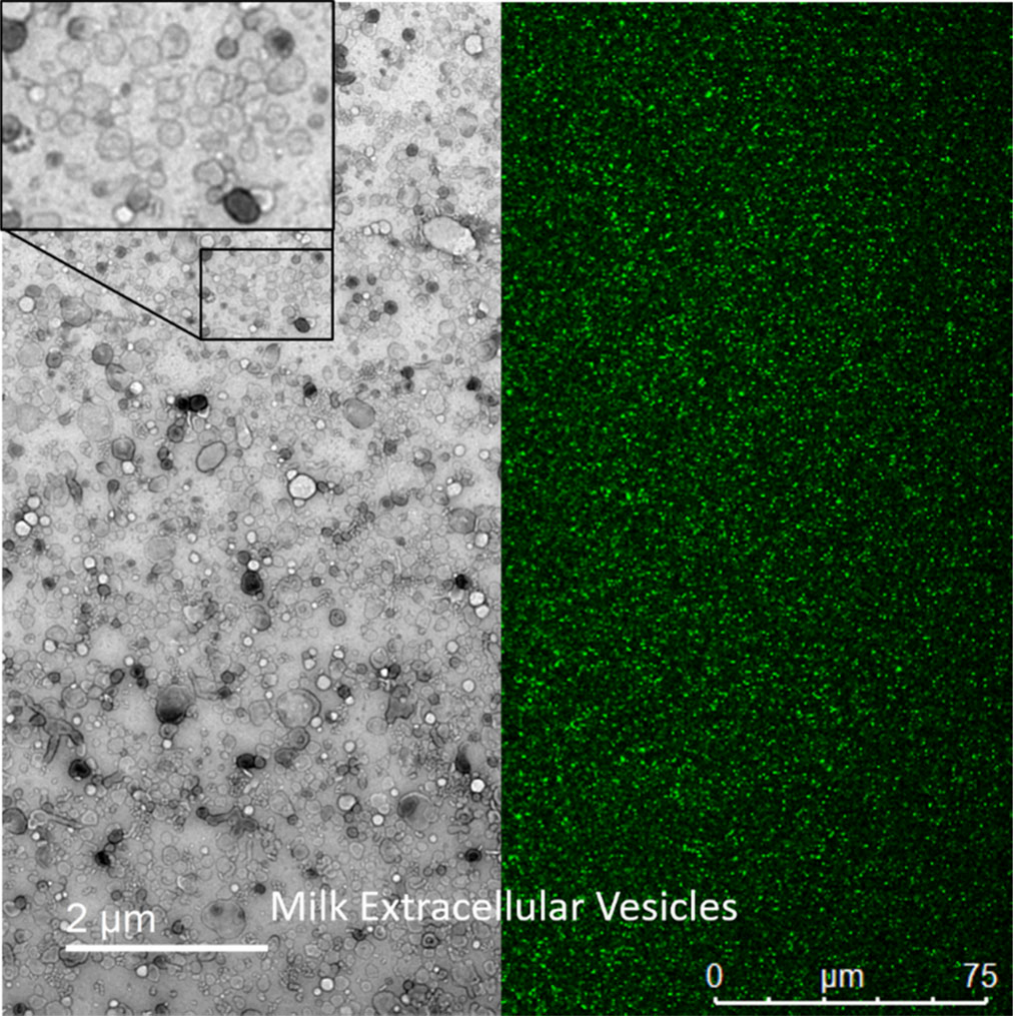
Representative transmission electron microscopy image of milk extracellular vesicles with high magnification inset (left) and Calcein‐AM‐stained milk extracellular vesicles imaged by confocal microscopy (right) repurposed with permission from authors.^1^

Additional characterization assays include Nanoparticle Tracking Analysis (NTA), which uses Brownian motion to track and trace EVs in order to determine the average size and particle density in a solution—multiple machines are used to accomplish NTA, with the most common being the NS500 from Malvern Panalytical.^90^, ^91^, ^92^ Owing to their size, often below the resolution limit of light microscopes, visualization of small EVs with visible light (380–700 nm) can be problematic. This being said, transmission electron microscopy is a popular method of direct visualization of EVs.^93^, ^94^, ^95^, ^96^ Scanning electron microscopy may also be used; however, the overall clarity and quality of the images tends to be of lower quality^97^, ^98^ Groups have also reported additional methods of characterization, including zeta potential measurement,^99^ fluorescent dye loading,^1^ and single molecule localization microscopy.^100^ Our own group has further developed assays based on the uptake and retention of esterified Calcein‐Acetoxymethyl (AM) in mEVs—a method that combines indirect visualization and an assay of bioactive esterase present in these nanoparticles—representative images repurposed with permission from authors from prior work are shown in Figure 3.^1^

## 3 BIOACTIVE CONSTITUENTS OF MILK EXTRACELLULAR VESICLES

mEVs have shown significant effects in preclinical testing in cell and animal models.^6^, ^11^, ^12^, ^43^, ^44^, ^45^, ^46^, ^101^ Importantly, the evidence suggests that mEVs provoke limited immune reaction, can be orally administered, and navigate their way readily through biological barriers within the body (e.g., blood–brain barrier) to provide system‐wide effects—with minimal losses resulting from filtration via the liver or kidneys.^47^, ^48^ As summarized in the text below and in Table 1, mEVs have shown notable bioactivity in infant development, particularly in postnatal maturation of neural, gut, and immune systems, as well as potential for mitigation of disease processes affecting various organ systems, including gastrointestinal, cutaneous, neurological, cardiovascular, musculoskeletal, and immunological cells and tissues.^6^, ^12^, ^43^, ^44^, ^45^, ^46^, ^98^, ^101^ These effects are typically linked to factors that modulate gene expression; indeed, mEVs are known to carry microRNAs, messenger RNAs, long‐non‐coding RNAs, and other types of genetic snippet.^44^, ^101^, ^102^ Impacts from RNAs are well documented; for instance, miR‐22‐3p is commonly found in mEVs, and has a known role in genetic repression of inflammatory signaling and the promotion of stem cell differentiation. Also notable is miR‐148a‐3p, which modulates DNA methyl‐transferase 1 expression—suggesting that recurring exposure to mEVs may have an impact on human genome epigenetics.^103^ The most common microRNA (miRNA) reported in mEVs include miR‐148a‐3p, miR‐30, miR‐146a and miR146b, miR‐200a, and miR‐200c, miR‐21.^44^, ^102^, ^104^, ^105^, ^106^ MicroRNA‐148a‐3p has consistently been identified as the most abundant miRNA found in mEVs—up to 24% of all mEV miRNA—as identified in original research and summarized in multiple reviews, highlighting the role of this RNA in mEV signaling, and potentially in epigenetic signaling.^103^, ^105^, ^106^, ^107^

**TABLE 1:** Nutraceutical uses of milk extracellular vesicles (mEVs) with primary investigator, citation number, assessment model, and a summarized major finding.

Primary investigator	Citation	Assessment model	Major finding
Janos Zempleni	6	C57ZBL/6 mouse	mEVs bypass gut blood and blood–brain barriers; mEV miRNAs in milk decreased seizure instance and increased neurological function (memory, spatial, and learning)
Thomas Thymann	10	Preterm Danish Landrace piglets	mEV treatments were associated with increased sphingolipids and odd‐chain lipids in brain tissues. No difference in memory or hippocampal lipid composition found
Agostino Pierro	12	IEC‐18 intestinal epithelial cells	Rat mEVs promoted IEC‐18 viability, enhanced proliferation, and stimulated intestinal stem cell activity
Yong‐Liang Zhang	14	Kunming mice	Pig mEVs enhanced villus height and crypt depth in Kunming mice, and enhanced expression of cvell proliferation associated genes CDX2, PCNA, Insulin Growth Factor‐1 Receptor (IGF‐1R), and decrease p53
Shuping Han	18	Human mEV peptide analysis	Preterm versus term mother breast mEVs analyzed; 47 peptides upregulated, 23 downregulated. Altered peptides known for regulatory roles in intestinal function
Agostino Pierro	11	Human mEV use on mouse pups	Raw and pasteurized mEVs improved NEC‐induced mucosal injury and inflammation; improved NEC‐altered mucous production
Gail E Besner	19	Rat pups‐induced NEC	Enteral administration of human mEVs decreased induced NEC incidence by >40%.
Marca Wauben	20	Industrial processing of bovine mEVs	Regular industrial processing approaches damages mEVs; ultra‐heat treatment destroyed mEVs, while standard pasteurization were found to disrupt membrane integrity and reduced mEV‐associated RNAs
Bodo C Melnik	21	Intestinal‐specific Kindlin‐2 Knockout mouse	Tamoxifen‐induced ulcerative colitis was prevented by oral gavage of bovine mEVs; treated mice displayed non‐inflamed intestinal mucosa
Regine Golan‐Gerstl	22	DSS‐induced colitis mouse	Cow and human mEVs attenuated severity of colitis and reduced expression of Interleukin‐6 (IL‐6) and Tumor necrosis factor‐α.
Janos Zempleni	25	C57Bl/6 mice	Cow mEVs altered microbiome in mouse gut; enhanced 52 independent taxonomic units (3 phyla, 7 families)
Huaxi Yi	43	DSS‐induced colitis mouse	Cow mEVs prevented colon shortening, reduced intestinal epithelium disruption, inhibited inflammatory cell infiltration and tissue fibrosis in a UC model
Jinquan Li	28	miRNA analysis of mEVs	37 miRNAs upregulated in mEVs versus milk; notable increases by miR‐193a‐3p, miR‐423‐5p, miR‐551a, miR‐138, miR‐1, and miR‐124a
Fons A J van de Loo	50	DBA/1J mice	Orally delivered cow mEVs increased osteocyte number, increased woven bone formation, decreased collagen; had temporal effect on bone mineralization
Janos Zempleni	51	C57Bl/6 mice	Cow mEVs resulted in moderate enhancements in gene expression and grip strength in skeletal muscle
Andreas N Kavazis	45	Fisher 344 rats	Depletion of cow mEVs in diet resulted in increased muscle fiber cross‐sectional area
S Oh	52	Glucocorticoid‐induced osteoporosis mice	Cow mEVs enhanced bone mineral density and restored gut microbiota community
Soraia Macari	53	Diet‐induced obese/ovariectomy mice	Orally administered cow mEVs protected from bone loss caused by obesity; ovariectomy induced increased osteoclast number, which was inhibited by mEVs
Sang Hun Lee	54	Sprague–Dawley rat pups	Orally administered mEVs enhanced long bone growth and increased bone mineral density of tibia
Jingfang Xiao	30	Isoproterenol‐induced cardiac fibrosis rats	Cow mEVs alleviated Extracellular Matrix deposition and enhanced cardiac function in cardiac fibrosis rat model. mEVs significantly increased angiogenic growth factors
Ji‐Young Ahn	32	RAW264.7 cells; IEC‐18 cells	Cow mEVs reduced LPS‐induced inflammatory signals (IL‐6, COX‐2, and nitric oxide) in RAW264.7 cells
Sun Hwa Kim	33	Keratinocytes, melanocytes, fibroblasts	Cow mEVs prevented Ultraviolet (UV)‐induced generation of intracellular reactive oxygen species in keratinocytes, melanin productions in melanocytes and suppressed Matrix Metalloproteinase production in fibroblasts

Abbreviation: NEC, necrotizing enterocolitis.

The nucleotide sequence‐based cargoes in mEVs vary considerably from those of other sources, such as from stem cells or other cell‐derived sources; this is further supported by the fact that healthy cow miRNA expression varies from diseased cows.^108^ Indeed, the health of the source, as well as the cellular source of EVs appears to have profound impacts on the cargoes held within EVs, further supporting continued research into EVs derived from developmental biofluids such as milk.^109^, ^110^

While cargoes targeting transcriptional processes such as miRNAs have previously garnered the most interest due to their effects in a number of models, increasing interest is being paid to the proteins found in mEVs. mEVs contain a wide range of polypeptides—indeed, the proteome of mEVs has been found to contain thousands of proteins,^111^ with variances in composition related to the age of the host, lactation stage, diet, species, and physiological health or illness.^48^, ^92^ Importantly, purification processes also influence the characterization of proteosomal cargos, heightening the relevance of using and reporting in detail any deployment of a MISEV‐approved purification procedure or standardized combination of procedures.^56^ The most abundant proteins identified in mEVs were summarized by Babaker et al.^112^ They found that mEVs contain proteins related to metabolism, protein homeostasis, immune response, endocrine functions, vesicle trafficking, as well as other biological processes. Importantly, it was determined that the concentration of different proteins varied depending upon the species, with bovine milk having a greater abundance of milk fat globule membrane proteins, while human mEVs showed higher levels of HSP70. With proteomes varying widely across species and EV types, research has focused primarily on proteomic analysis. In contrast, the lipidome is generally viewed as a consistent component of EVs.

## 4 BIOAVAILABILITY OF MILK EXTRACELLULAR VESICLES FOLLOWING ORAL ADMINISTRATION

Research findings have highlighted the remarkable efficacy of purified mEVs upon oral ingestion, showcasing their targeted distribution and therapeutic potential across diverse organs and tissues.^47^, ^113^, ^114^, ^115^, ^116^, ^117^, ^118^, ^119^ Studies have demonstrated the ability of mEVs to traverse the gastrointestinal tract intact and reach the systemic circulation,^47^, ^113^, ^114^, ^115^, ^116^, ^117^, ^118^, ^119^ subsequently targeting organs including lungs, heart, kidneys, liver, spleen, brain, and placenta^47^, ^113^, ^114^, ^115^, ^116^, ^117^, ^118^, ^119^; further revealing mEV uptake in cells and tissues within hours of oral ingestion. Specifics regarding these studies are summarized in Table 1. In one study, Manca et al. assessed the bioavailability and distribution of mEVs and their miRNA cargos from mouse, cow, and pigs within and between species boundaries.^47^ Fluorescently labeled mEVs were found at elevated densities in numerous organs, including the liver, spleen, and brain following suckling, oral gavage, and intravenous administration in mice and pigs. Interestingly, this study provided hints that biodistributions achieved by oral versus intravenous routes may differ in subtle, but important ways.^47^ These findings underscore the versatility and longevity of milk EV bioactivity following oral ingestion, offering intriguing prospects for the development of novel therapies, with implications for various health conditions, including liver disorders, immune‐related diseases, and neurological disorders.

## 5 EFFECTS OF NATIVE MILK EXTRACELLULAR VESICLES ON DEVELOPMENT, MATURATION, AND DISEASE

With cargoes that can target gene expression and/or possess ligand‐like or enzymatic activities, EVs represent potent vehicles for delivering both short‐ and long‐range messages between cells and tissues within the body as part of natural biological processes. mEVs are evolutionarily designed to: (1) be orally consumed—a unique aspect of mEVs versus other sources of EVs; (2) efficiently bypass the gut‐blood barrier,^120^ show reduced propensity for filtration by the liver and kidneys,^47^, ^48^ (3) show a low level of immunogenic provocation,^119^ and (4) bypass a variety of tissue boundaries, including the blood–brain barrier.^6^ With growing understanding of their potential to impact the development and overall health and wellness of infants, mEVs have been subject to intensive study since their emergence as a topic of research over the last decade.^6^, ^11^, ^12^, ^43^, ^44^, ^45^, ^46^, ^101^ The fruits of this labor are a growing understanding that mEVs may influence virtually every organ system in the developing infant. Whilst their effects are most notable in newborns, the potential for benefit is evident across the human lifespan, encompassing diverse processes including neurological development and musculoskeletal repair following exercise.^6^, ^11^, ^12^, ^43^, ^44^, ^45^, ^46^, ^101^ In particular, the recent uncovering of possible impacts on infant brain and cognitive development is of special interest to parents around the world.

### 5.1 Milk EV effects on the brain and cognition

Some of the most intriguing effects noted in studies performed in vivo have been the characterization of effects on infant brain development. In one prominent study, Zhou et al. assessed the ability of mEVs to bypass the gut blood and blood–brain barriers.^6^ Results indicated that mEVs bypass all major internal barriers, including the blood–brain barrier, and influenced brain development when present in mouse pups' diet. When comparing pups given standard breast milk versus those given mEV mRNA‐depleted milk, dramatic differences were noted in brain development and neurological function. Pups without mEV miRNAs had reduced dendritic complexity of dentate granule cells in the hippocampus, experienced five‐fold more severe seizures following kainic acid challenge, and scored nine‐fold lower in a classic Barnes Maze test of spatial learning and memory. This study illustrates not only the ability of mEVs to reach tissues throughout the body following oral administration, but more importantly, the profound impact that their cargoes may have on health and development, particularly in the neurological system of developing infants.

In contrast, findings from Henriksen et al.^10^ presented a more nuanced perspective. Their investigation, conducted in preterm piglets, evaluated the impact of formula diets enriched with either EVs or phospholipids over a 19‐day period. Analysis of brain and plasma samples revealed significant variations in fatty acid composition, albeit with minor distinctions observed between the EV and phospholipid groups. Behavioral assessments, including novel object recognition and T maze tests, revealed subtle differences between experimental cohorts, with no statistically significant variations detected. Importantly, the study highlighted alterations in plasma lipid levels and hippocampal tissue diffusivity attributed to both EVs and phospholipids, though without observable effects on memory function. It is noteworthy that commercially sourced EVs were utilized in this investigation, with no detailed information provided regarding the isolation methodology or characterization data for the EV isolates. These findings underscore the complexity of interpreting the effects of EVs versus phospholipids on physiological parameters and behavioral outcomes, warranting further research elucidating the mechanisms underlying their differential impacts.

The context of studies comparing the neurological development of breastfed and formula‐fed infants may provide valuable insight into this debate, particularly given that formula may represent an EV‐depleted diet due to the processing methods commonly employed by the dairy industry, such as air drying at 200°C to produce milk powders, which can adversely affect lipid vesicle structure. Consistent comparisons between these two feeding regimes reveal notable advantages for breastfed infants, who tend to perform better on tests assessing mental and psychomotor development.^7^, ^8^ Additionally, research indicates that breastfeeding positively influences brain structure, leading to increased volumes of white matter, subcortical gray matter, and cortical thickness in infants. A meta‐analysis further supports these findings, showing that breastfed infants score higher on cognitive assessments compared to their formula‐fed counterparts.^9^ However, it is essential to exercise caution in attributing causality to the constituents of EVs in breast milk based on these studies—such definitive conclusions cannot yet be drawn. See Table 1 for a summary of the impacts of mEVs on the brain and cognitive function.

### 5.2 Milk EV effects on the gastrointestinal system

Much like the growing body of work on the brain and nervous system, impacts of mEVs have been found on gastrointestinal development, as well as in the mitigation and treatment of gastrointestinal diseases. These studies began with early investigations into the role of mEVs performed in vitro using intestinal epithelial lines, such as IEC‐18 and IEC‐J2 cells.^13^, ^29^ Results indicated the ability of mEVs from porcine^14^ and murine^12^ sources to promote cell proliferation, migration, viability, and stem cell activity. Transcriptional analysis also indicated changes in expression of CDX2, IGF‐1R, and PCNA coupled with inhibition of p53 expression, markers that are correlated with proliferation of intestinal cells and tissues.^14^ Results from these experiments were among those that launched a new phase of milk EV research, using in vivo models of gastrointestinal development and disease.

From 2019 to 2020, three groups published results studying the effects of mEVs on gut maturation and on necrotizing enterocolitis (NEC). NEC is the leading cause of gastrointestinal disease‐related death in preterm infants, affecting 5%–12% born at a very‐low birth weight.^15^ Indeed, mortality rates for infants with NEC are 10%–50%, depending upon birth weight^15^, ^16^, ^17^; crucially, there has been no significant change in these rates in the last 20 years, highlighting a need for new therapeutic approach.^16^ For further reading, a review summarizing the efficacy of mEVs in treating NEC has been published.^17^ In one of the first studies in the primary literature, Wang et al.^18^ analyzed breast milk samples from healthy lactating mothers who had delivered either term or preterm babies. Following exosome purification from these milk samples, the peptidomic compositions were analyzed; a total of 70 peptides were found to be significantly changed, with 47 upregulated and 23 downregulated. Bioinformatic analysis of peptides undergoing change suggested potential for effects in several biological domains, supporting the need for further research on EV composition in mothers giving birth prematurely.

Following this publication, Pisano et al.^19^ determined that EVs isolated from human donor breast milk, depending upon administration path, were capable of reducing experimentally induced NEC incidence in animal models to less than 12% (*p* < 0.05). The primary findings from Pisano suggested that breast mEVs, regardless of administration method, significantly decreased the incidence and severity of NEC. A month later, Miyake et al.^11^ confirmed this phenomenon by providing a report on the effects of exosomes purified from human breast milk on mouse pups with induced NEC. Interestingly, Miyake et al. determined that pasteurization of human breast milk did not impact the effects of this exosomal treatment, suggesting mEV stability under pasteurization conditions. Other groups have published results that seemingly dispute this notion, suggesting more work is needed.^20^ The primary conclusions by Miyake indicated that exosomes isolated from human breast milk are capable of decreasing inflammation, improving mucous production, and reducing NEC‐induced intestinal injury.

Further in vivo experiments published in 2020 analyzed the effect of mEVs on ulcerative^21^ and dextran sulfate sodium (DSS)‐induced colitis.^22^ First, Stremmel et al.^21^ reported that mEVs isolated from commercial bovine milk had cytoprotective and anti‐inflammatory activities in an intestinal‐specific Kindlin‐2 knockout mouse model of ulcerative colitis. Cow mEVs were provided in one dose of 33 μg/g of body weight. In response, a reduction in disease severity was observed, as well as significant increases in colon weight and length and improvement in stool appearance. Reif et al. published results in late 2020 on the impact of mEVs on DSS‐induced colitis.^22^ Mice were treated with DSS to induce colitis and then were provided with orally administered mEVs or control treatments. Following proteomic and transcriptomic analyses of the Gastrointestinal (GI) tract, it was found that the EVs delivered miRNA cargoes to intestinal cells, and demonstrated overall therapeutic and anti‐inflammatory effects on the GI tract. These studies, in conjunction with those of previous groups, suggest that naturally occurring mEVs from bovine, human, porcine, and even commercial sources are effective at treating infantile GI diseases, such as NEC and ulcerative colitis. See Table 1 for a summary of the impacts of mEVs on the gastrointestinal system.

### 5.3 Milk EV effects on the gut microbiome and immune system

In addition to having profound effects on the gastrointestinal system, mEVs have been shown in preclinical studies to impact the immune system and microbiome of recipient animals. Commercial products to support healthy microbiome development have elicited renewed interest in recent years; indeed, forecasts suggest that the commercial market for microbiome‐based therapies will reach $1.3b by 2030.^23^, ^24^ In 2019, Zhou et al.^25^ reported on the effects of mEVs provided in either mEV/RNA‐depleted or mEV/RNA‐sufficient diets based on AIN‐93G formulation.^26^, ^27^ These results indicated that mEVs altered the bacterial load in the murine cecum, with 52 operational taxonomic units experiencing significant differences from mice not provided mEVs. These data indicate that bovine mEVs may alter microbial communities in non‐bovine species, suggesting that mEVs are capable of promoting communication between bacterial and animal kingdoms, a groundbreaking finding.

Similar to previous experiments showing the impact of mEVs on treating and mitigating colitis in a number of models (DSS‐induced, etc.), Tong et al. reported in 2021 that the mechanism by which mEVs impart their beneficial effects in colitis was via regulation of the gut immune system and microbiome.^43^ Using the DSS‐induced model,^22^ these authors showed that the proteins and RNAs present in EVs from milk were directly involved in the regulation of both immune and inflammatory pathways. Additionally, they confirmed that following oral administration, mEVs prevented colon shortening, inhibited inflammatory cell infiltration and tissue fibrosis, and reduced barrier function disruption of the intestinal epithelium in a DSS‐induced mouse model of ulcerative colitis. Of note, the disturbed microbiota in this disease model was partially recovered following treatment with EVs derived from milk, suggesting that intestinal immunity is modulated by mEVs via regulation of the gut microbiota.

Following these discoveries, Liu et al.^28^ published an in‐depth characterization of milk exosomal microRNAs, in an attempt to determine the role and function of these transcriptional inhibitors. It was found that specific miRNAs were upregulated more than a 1000‐fold in mEVs as compared to starting milk samples, and that these miRNAs appeared to be critical to cell development and basic physiological maintenance of the immune system. Following gene sequencing, 9262 target genes were identified that were concentrated on three major pathways: metabolic signaling, cancer, and PI3K/Akt signaling. Further in‐depth analyses of the pathways identified that the exosome‐concentrated miRNA target genes were specifically involved with metabolism and immunity, further strengthening arguments that mEVs play a crucial role in the maturation of immune function in infants, as well as highlighting that purified and concentrated doses of mEVs may be required for optimizing certain types of desired biological activity. See Table 1 for a summary of the impacts of mEVs on the gut microbiome and immune system.

### 5.4 Milk EV effects on muscle and bone

While milk has long been thought to have beneficial effects on musculoskeletal growth and function,^49^ recent work has also suggested roles for mEVs in bone development. Early studies in this field were performed by Oliveira et al.,^50^ who determined that bovine mEVs markedly increased woven bone formation, osteocyte number, and proliferation. Interestingly, mEVs reduced collagen production, yet enhanced the expression of genes associated with immature osteoblasts. The authors acknowledged that while some findings on gene expression were unexpected, the study overall supported further investigation into mesenchymal extracellular vesicles (MSEVs) and their potential to promote bone formation in both infants and adults.

In a study published in 2018 by Leiferman et al.,^51^ the multifaceted impact of mEVs on musculoskeletal health was probed using an in vivo mouse model. Exosomes isolated from bovine milk were administered to C57Bl/6 mice maintained on an AIN93G diet, either devoid of exosomes or supplemented with a full complement of EVs. The administration of mEVs resulted in discernible yet moderate enhancements in gene expression and grip strength within skeletal muscle. However, the observed effects appeared comparatively subdued when juxtaposed with effect sizes reported in other studies, prompting inquiry into the nuanced mechanisms that may underlie the influence of mEVs on musculoskeletal tissues. Notably, the investigation by Parry et al.^45^ shed further light on this nuance by demonstrating that rats deprived of EVs in their AIN‐93G diet exhibited significantly increased muscle fiber cross‐sectional area following 28 days of feeding compared to counterparts receiving a full complement of EVs. These findings suggest that mEVs orchestrate intricate and context‐dependent effects on skeletal muscle growth and maturation.

Studies on mEVs in relation to bone suggest larger effects than those reported for muscle. Expanding upon previous work investigating the impact of mEVs on bone development, Yun et al.^52^ determined that EVs from milk promote an anti‐osteoporosis phenotype in an in vivo model of glucocorticoid‐induced osteoporosis. EV‐treated groups experienced increased bone mineral density, as well as enhanced gut microbiota. Importantly, this study used a pre‐treatment of mEVs for 2 months prior to the induction of osteoporosis, suggesting that mEVs are functional as a prophylactic treatment for osteoporosis. Meanwhile, Oliveira et al.^53^ provided mEVs in the drinking water of mice in two separate models of bone loss: obesity or ovariectomy. Mice who received mEVs were protected from bone loss caused by diet‐induced obesity, while those receiving an ovariectomy experienced higher osteoclast numbers in the femur, which was mitigated by treatment with EVs. In addition, reduced femur stiffness induced by ovariectomy was rescued with EV treatment. This research illustrates the impacts that mEVs may have on bone structure and function, potentially serving as a therapeutic candidate in different types of pathologic bone loss. Recent work by Go and co‐workers further demonstrated that oral administration of mEVs to Sprague–Dawley rat pups resulted in enhanced long bone growth and increased bone mineral density of the tibia, suggesting a crucial role of mEVs in bone development.^54^ See Table 1 for a summary of mEV impacts on bone and muscle.

### 5.5 Milk EV effects on the cardiovascular system

Whilst work elucidating assignments of mEVs in the cardiovascular system has been limited to date, Zhang et al.^30^ showed in 2021 that in an isoproterenol‐induced murine model of cardiac fibrosis, exosomes from milk alleviated excess extracellular matrix deposition and enhanced cardiac function. Specifically, pro‐angiogenic factors were significantly enhanced in rats treated with mEVs. Though this is one of the few examples to date showing an effect of mEVs on cardiac function, there are a number of groups advancing cardiac therapies using mEVs as a drug delivery vehicle—a key focus in the field of mEVs. See Table 1 for a summary of mEV impacts on the cardiovascular system.

### 5.6 Milk EV effects on skin

As summarized in the previous sections, small EVs isolated from milk have been shown to be adept at beneficially modulating injury and inflammatory processes in various tissues. Cutaneous tissues provide further examples, with recently published reviews covering knowledge in the field.^31^ Specifically noted is the ability of exosomes to provide therapy against damage from UV light, infrared radiation, and burn wounds. There are reports that mEVs may promote scar‐free healing in an in vitro analysis using IEC‐18 cells,^29^ by increasing transforming growth factor‐β3 activity and reducing inflammation.^32^ Additional in vitro research has indicated that bovine mEVs have the potential to promote repair of UV‐irradiated dermal tissues.^33^ Research into dermal wound healing has, to date, largely been limited to in vitro analyses, highlighting the need for further research into the effects of mEVs on cutaneous wound healing using in vivo models. See Table 1 for a summary on the impacts of mEVs on skin healing.

## 6 CLINICAL USE OF MILK EXTRACELLULAR VESICLES AS NUTRACEUTICAL ADDITIVES

Despite the growing base of evidence that mEVs serve important roles in development, maturation, and treatment of disease, publications on clinical trials utilizing mEVs as a nutraceutical additive are yet to emerge. This is confirmed through conversations in the field as well as searching for any associated keywords in Clinicaltrials.gov. The clinical translation of mEVs is hampered by the lack of any Food and Drug Administration (FDA)‐approved, Chemical and Manufacturing Control (CMC)‐validated facility that can produce mEVs at the levels required for large studies. To date, only one group has shown the capability to mass produce mEVs,^55^ which was a pilot study done at non‐Good Manufacturing Practices (GMP) conditions. This lack of industrial, GMP‐grade manufacturing has limited mEVs from entering clinical testing in humans. This being said, it seems likely that GMP production of mEVs is a hurdle that will soon be cleared. A unique aspect of mEVs that has stymied industrial production is the fact that the specific cellular source of mEVs has not been confirmed, so the starting solution is a complex biofluid with fats and a matrix of casein and whey proteins, which requires innovative new approaches to purification versus standard bioreactor‐based production of cell‐derived EVs, such as mesenchymal stem cell extracellular vesicles (MSEVs). A further barrier to clinical translation is the lack of thorough pharmacokinetic studies that investigate Absorption, Distribution, Metabolism and Excretion (ADME)—mechanistic data that is required for entry into FDA‐approved clinical trials. Many studies have utilized fluorophore‐based tracking of mEVs, but this is generally not considered sufficient for ADME characterization.

Given that other types of EVs have already entered clinical testing, one might wonder why it is worthwhile to explore the translation of mEVs into clinical trials instead of utilizing other sources, such as MSEVs, which are currently employed in a number of active and completed clinical trials.^121^, ^122^ However, mEVs present unique advantages that merit consideration, including their natural biocompatibility, scalability, and cost‐effectiveness. Additionally, mEVs contain a diverse array of bioactive molecules that have evolved to support growth and development, and thus may offer potential for oral delivery due to their role in the digestive system. These factors suggest that mEVs could serve as a valuable complement to existing EV sources in therapeutic applications. This is primarily due to the unique nature of mEVs noted above, such as their (1) ability to be orally administered, (2) propensity to avoid liver filtration,^47^, ^48^ (3) gut‐blood barrier bypass,^120^ (4) blood–brain barrier bypass,^6^ and (5) relatively non‐immunogenic nature,^119^ being a few of the reasons as to why mEVs are a desirable form of EV to advance to clinical testing. Once mEVs are produced under GMP conditions and can be reliably manufactured at industrial levels, their study in clinical trials seems likely to follow.

## 7 MILK EXTRACELLULAR VESICLES AS PHARMACEUTICAL CARRIERS

**FIGURE 4:**
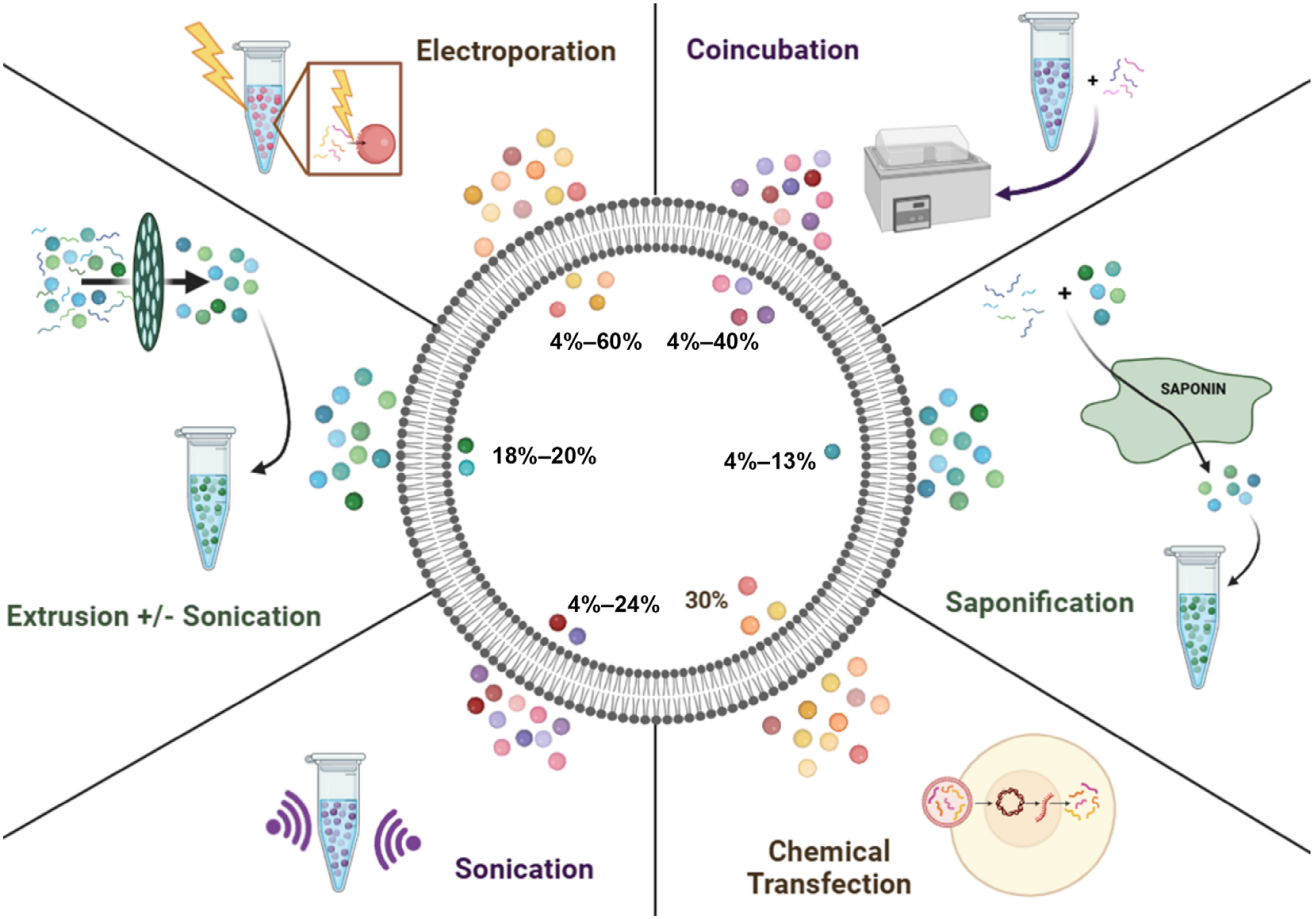
Overview of loading methods leveraged to date for mEVs. Electroporation^42^ and co‐incubation^34^, ^37^, ^39^ have shown the greatest loading ceiling (40%–60%), with notable floors of 4%.^35^ Chemical transfection has shown a consistent result of 30%,^35^ while other approaches, including sonication^36^ and saponification,^41^ have shown low efficacy. Combination approaches such as extrusion + sonication^40^ have shown improvements over sonication alone,^41^ but with notable limits on efficacy. See Table 2 for an itemized breakdown of loading efficiencies and cargoes utilized, as well as the specific citations associated with the methods summarized.

EVs have thus been shown to be potent carriers of naturally occurring, and in some cases, nutraceutical cargoes. This is due to a number of beneficial variables, including low or non‐immunogenicity and stability in the gut. Given that mEVs have shown potent impacts throughout the body, a major focus in the field has been on developing methods to functionalize, load, and utilize mEVs as a drug delivery platform, carrying exogenous therapeutic molecules to tissues and cells of interest within the body. A number of groups have published work reporting the loading of mEVs with medicinal cargoes, including RNAs, small molecules, peptides, plasmids, and more.^34^, ^35^, ^36^, ^37^, ^38^, ^39^, ^40^, ^41^, ^42^ See Table 2 for a comprehensive summary of literature published to date regarding the use of mEVs for the delivery of pharmaceutical agents. See Figure 4 for an illustrative summary of the methods used and their relative efficiencies for loading mEVs with exogenous drug cargoes.

**TABLE 2:** Pharmaceutical use cases of milk extracellular vesicles with primary investigator, citation number, drug loaded, method of drug loading and efficiency of drug loading.

Primary investigator	Citation	Drug loaded	Loading method	Efficiency noted
Ramesh C. Gupta	34	Paclitaxel, Wirthaferin A	Co‐incubation	10%–40%
Ramesh C. Gupta	35	Small interfering RNA (siRNAs); si‐VEGF, si‐EGFR, si‐AKT, si‐MAPK, and si‐KRAS	Electroporation; chemical transfection	4%–5% by electroporation 30% by transfection
Zhenbing Chen	36	siRNAs; si‐Keap1	Sonication	Up to 24%
Zhimeng Wu	37	Doxorubicin	Co‐incubation	21.2%
Youxin Li	38	Liraglutide	Co‐incubation versus extrusion	Extrusion achieved 2.45× direct incubation
Xiaomei Yan	39	Doxorubicin	Sonication and Extrusion‐assisted Active Loading “SEAL”	18.2%
Vengala Rao Yenuganti	40	Doxorubicin	Incubation, saponification, sonication	Incubation: 4%–13% Saponification: 4%–13% Sonication: 4%–12%
Xiang Xu	41	miRNA; miR‐31‐5p	Electroporation	>60%

### 7.1 Milk extracellular vesicles delivering anti‐cancer agents

The first publication in the field was published in 2016 by Munagala et al.,^34^ outlining a method to put functionally active cargoes into mEVs. In this study, Munagala and colleagues loaded anti‐cancer drugs, including Paclitaxel and Withaferin A and delivered them in vivo, demonstrating that drug encapsulation within mEVs is a practical and effective route forward. These findings laid a foundation for ongoing research, establishing a proof‐of‐concept for treatment of disease with mEVs carrying exogenous cargoes.

### 7.2 Milk extracellular vesicles delivering exogenous RNA cargoes

The next report on the effects of drug‐loaded mEVs was by Aqil et al.^35^; both Aqil and Munagala are part of the Gupta lab, so this research was an extension of that cited above.^34^ In these studies, Aqil et al. attempted to load mEVs with different siRNAs for delivery to recipient cells. They found variable efficiencies of their approach, depending upon the siRNA and target gene, with efficiency ranging from 2 to 10‐fold knockdown in expression levels. Conclusions from this work included that siRNA is able to be loaded and delivered to target cells by EVs, providing supporting evidence for the contention that these nanovesicles may be a clinically effective carrier for siRNAs in the treatment of disease, especially cancers. More specifically, it was found that chemical transfection was over six times more efficient in loading siRNAs than electroporation, supporting transfection as a more effective loading approach than electroporation. In addition to Munagala, Xiang et al.^36^ confirmed the ability of mEVs to be loaded with siRNA and deliver such cargos to cells in vivo. In order to knock down Keap1, targeting siRNAs were put into mEVs using a sonication‐based protocol. When tested in human umbilical cord endothelial cells, siRNA‐Keap1‐loaded mEVs promoted migration and proliferation, while relieving oxidative stress. In a mouse model of diabetic wounds, treatment with siRNA‐Keap1 mEVs resulted in an acceleration of wound healing, with enhanced neovascularization and collagen formation.

### 7.3 Modulation of the MEV membrane to enhance targeting capabilities

Further research into the utilization of mEVs as a pharmaceutical carrier has involved decorating the surface of mEVs with various moieties in order to direct delivery to specific cells and tissues, primarily to enhance targeting of cancer cells and tumors. To this end, Li et al.^37^ attempted to develop a novel strategy to direct doxorubicin‐loaded mEVs to CD44‐overexpressing tumor cells. To accomplish this, these workers decorated the surface of mEVs with hyaluronan, a CD44‐specific ligand, which was then functionalized with an amphiphilic molecule, DSPE‐PEG2000. This functionalization enabled the spontaneous decoration of the phospholipid bilayer. The final nanocarrier, a hyaluronan‐labeled mEV loaded with doxorubicin, then was able to selectively deliver doxorubicin into cancer cells overexpressing CD44 and trigger cell death. Notably, this study was performed in vitro in cultured cells. Further research is required to determine the ability of decorated mEVs to traverse the internal environment and effectively target tissues in vivo.

Other research on functionalizing the surface of mEVs to optimize drug delivery to tissues of interest includes that reported by Go et al.^38^ These authors utilized oxaliplatin as the chemotherapeutic cargo and GE11 peptide as the targeting moiety conjugated to the EV surface. GE11 peptide has a high affinity for epidermal growth factor receptor (EGFR), enabling tumor targeting. Results from these experiments determined that oxaliplatin‐loaded EVs conjugated with GE11 peptide showed significantly higher incorporation into EGFR‐expressing cancer cells compared to those without GE11 peptide conjugation, leading to increased apoptosis of cancer cells. mEVs loaded with oxaliplatin and conjugated with GE11 showed the maximum therapeutic effect when compared with oxaliplatin alone or oxaliplatin‐loaded mEVs without GE11 conjugation. These results indicate that while drug loading is an important factor in using mEVs as a drug delivery platform, the effective decoration of mEVs with targeting moieties may be a crucial component to effective tissue and cell targeting for uses in oncology. This being said, an important question of such approaches is whether immune responses are activated against normally immune‐quiescent mEVs by purposefully engineering them for targeted behaviors in vivo.

### 7.4 Extrusion‐based loading of milk extracellular vesicles

More recently, Shi et al.^39^ demonstrated the ability of mEVs to deliver peptide drugs, namely, Liraglutide, a GLP‐1 receptor agonist. Six drug‐loading methods were tested, including freeze‐thawing, sonication, and direct incubation‐based approaches. It was found that the most effective method of promoting drug uptake was extrusion of EV solutions through 100 nm apertures, which resulted in 2.45× loading over direct incubation. These studies confirm the ability of mEVs to provide an oral route of administration for GLP‐1 agonists, which have gained increasing interest due to commercial products for diabetes and weight loss, such as Ozempic, becoming available in recent years. The opportunity of moving away from injected to orally available GLP‐1 agonist treatments appears to be a useful clinical advance that should be explored in coming years.

Extrusion loading of vesicles has not only been validated by Shi et al., but also by Chen et al.,^40^ who created a novel method named “Sonication and Extrusion‐assisted Active Loading,” or SEAL, for effective encapsulation of doxorubicin into mEVs. Chen and colleagues found that SEAL resulted in around a 10‐fold enhancement of drug encapsulation efficiency compared with passive loading. Importantly, these workers also determined that interfering protein micelles such as caseins were ineffectively loaded with doxorubicin, while only those EVs with intact cell membranes showed efficient loading with the drug. These results, coupled with data provided by Shi et al., suggest that drug loading using active interventions (e.g., extrusion) may be required to enhance drug‐loading efficiency of mEVs.

### 7.5 Comparison of mEV drug loading approaches

In a comparative study using numerous sources of mEVs, Ahmed et al.^41^ investigated the loading efficiency of doxorubicin into EVs from cow, buffalo, and goat milk using direct incubation, saponin treatment, and sonication. In all three species, mEVs were spherical with sizes <200 nm and expected exosome markers present, confirming that the isolation procedures used were appropriate and capable of purifying EVs from each of the sources studied. Conclusions from these studies indicated that goat EVs showed the highest loading potential across all three encapsulation methods assessed and resulted in the best drug delivery profile of each mEV type. Release profiles of doxorubicin by mEVs were biphasic, with an initial burst followed by a phase of more sustained delivery. These observations suggested that EVs from goat milk may be an opportune source for use in drug delivery. A further conclusion from these studies is that ongoing investigation may be required to determine the effects of species on the optimized use of mEVs for the delivery of exogenous therapeutic cargoes.

Further active loading approaches of mEVs include electroporation, as shown by Yan et al.,^42^ who loaded mEVs with exogenous miR‐31‐5p using an electroporation‐based approach in order to enhance wound healing in diabetic patients. It was then demonstrated that they not only were able to load miR‐31‐5p into mEVs, but that the miR‐31‐5p present in the vesicles was successfully protected and delivered to cells, resisting degradation. This exosomal therapeutic dramatically increased endothelial cell function in vitro, and, more importantly, promoted angiogenesis and enhanced diabetic wound healing in vivo. These data suggest that loading of exogenous miRNA cargos into mEVs is not only feasible, but results in a potent bioactive product capable of providing a therapeutic for accelerating diabetic wound healing via promotion of angiogenesis. See Table 2 below for a summary of the literature published to date regarding the use of mEVs as pharmaceutical carriers and Figure 4 for an overview of the methods used and their relative efficiencies for loading mEVs with exogenous drug cargoes.

## 8 CONCLUSIONS

A growing body of evidence underscores the remarkable bioactivity of mEVs to contribute to the health and well‐being of mammalian infants during postnatal development. While the sustained consumption of animal milk into adulthood has historically offered nutritional advantages to humans since the neolithic revolution and rise of agriculture, the nutraceutical potential of its EV constituents has until quite recently been unrecognized. Pertinently, modern dairy processing techniques, notably heat pasteurization, risk diminishing the bioavailability of these vesicular components in milk products consumed in developed nations—a recent departure from evolutionary norms. A burgeoning body of research indicating the potential nutraceutical benefits of mEVs across various mature tissues, including the gut and bone, underscores the urgency for innovative approaches in isolating and utilizing these vesicular components safely (Table 1). This entails the standardization of isolation methodologies on an industrial scale, alongside rigorous characterization of EV isolates to elucidate their structure, function, and bioactivity. Moreover, recent investigations have demonstrated the capacity of small EVs derived from milk to serve as efficient carriers of pharmaceutical agents such as small drug molecules, peptides, siRNAs, and miRNAs, opening avenues for further exploration in orally available nutraceutical product development and pharmaceutical delivery systems (Table 2). This ability to carry novel therapeutic cargoes includes the prospect of somatic delivery of gene‐editing modules, such as those based on CrispR‐Cas9, obviating the need for risky viral vectors for such purposes. Moving forward, sustained research efforts are imperative to fully harness the potential of mEVs, both as nutraceutical agents and as versatile pharmaceutical carriers, promising substantial advancements in both health promotion and therapeutic innovation.

## AUTHOR CONTRIBUTIONS

**Spencer R. Marsh:** Conceptualization; writing – original draft; funding acquisition; writing – review and editing; project administration; supervision. **Claire E. Beard:** Data curation; investigation; writing – review and editing; visualization; software. **Robert G. Gourdie:** Project administration; supervision; writing – review and editing; conceptualization; funding acquisition.

## CONFLICT OF INTEREST STATEMENT

SRM and RGG are company officers at The Tiny Cargo Company, a corporation commercializing milk EV technologies.

### PEER REVIEW

The peer review history for this article is available at https://www.webofscience.com/api/gateway/wos/peer‐review/10.1002/btm2.10756.

## Data Availability

No primary data are provided; all cited data are available in the main manuscript or citations.
